# Search of inhibitors of aldose reductase for treatment of diabetic cataracts using machine learning

**DOI:** 10.1016/j.aopr.2023.09.002

**Published:** 2023-10-03

**Authors:** Trevor Chen, Richard Chen, Alvin You, Valentina L. Kouznetsova, Igor F. Tsigelny

**Affiliations:** a MAP Program, University of California, San Diego, La Jolla, CA, USA; b San Diego Supercomputer Center, University of California, San Diego, La Jolla, CA, USA; c CureScience Institute, San Diego, CA, USA; d Department of Neurosciences, University of California, San Diego, La Jolla, CA, USA

**Keywords:** Diabetic cataracts, Aldose reductase inhibitor, Machine learning, Deep learning

## Abstract

**Purpose:**

Patients with diabetes mellitus have an elevated chance of developing cataracts, a degenerative vision-impairing condition often needing surgery. The process of the reduction of glucose to sorbitol in the lens of the human eye that causes cataracts is managed by the Aldose Reductase Enzyme (AR), and it is been found that AR inhibitors may mitigate the onset of diabetic cataracts. There exists a large pool of natural and synthetic AR inhibitors that can prevent diabetic complications, and the development of a machine-learning (ML) prediction model may bring new AR inhibitors with better characteristics into clinical use.

**Methods:**

Using known AR inhibitors and their chemical-physical descriptors we created the ML model for prediction of new AR inhibitors. The predicted inhibitors were tested by computational docking to the binding site of AR.

**Results:**

Using cross-validation in order to find the most accurate ML model, we ended with final cross-validation accuracy of 90%. Computational docking testing of the predicted inhibitors gave a high level of correlation between the ML prediction score and binding free energy.

**Conclusions:**

Currently known AR inhibitors are not used yet for patients for several reasons. We think that new predicted AR inhibitors have the potential to possess more favorable characteristics to be successfully implemented after clinical testing. Exploring new inhibitors can improve patient well-being and lower surgical complications all while decreasing long-term medical expenses.

## Highlights

• Diabetic cataracts need a specific treatment related to inhibition of Aldose Reductase.
• Machine-learning model trained to find new Aldose Reductase inhibitors.
• Novel Aldose Reductase inhibitors have stronger binding affinity than benchmarks.
• Mitigate diabetic cataract using these inhibitors can improve patient outcomes and reduce medical costs.

## 1 Introduction

Cataract is a condition that degenerates vision due to a clouding of the lens.^1^ Aldose Reductase (AR) is an enzyme that primarily breaks down glucose into sorbitol.^1^ It is primarily present in the lens and retina, and when not inhibited correctly, it can release high amounts of sorbitol into the lens. These high levels of sorbitol are responsible for diabetic cataracts by causing hydropic lens fibers to degenerate, clouding up the lenses.^1^ Thus, by inhibiting AR, sorbitol levels are decreased, preventing or delaying the formation of diabetic cataracts. Various studies have shown that current Aldose Reductase inhibitors are effective and do prevent cataracts,^2^ however more can be done to create better inhibitors. Our project expands upon current research so we can elucidate new compounds that would prevent and treat cataracts.

By combining current data with machine learning (ML), a prediction model can be created to help find future, more effective and safer medicine for Aldose Reductase inhibition. Taking different characteristics of each type of known inhibitor, it is possible to create a model that will search compounds to find an inhibitor that has all the most important characteristics that would inhibit Aldose Reductase. Using this information, this model could be applied elsewhere to other types of similar conditions. We hope to expand on this possible pathway and prove its ability to become a reliable method.

Several groups used Machine Learning (ML) for prediction of new inhibitors of other proteins with the following testing by computational docking. Huang and colleagues described development of RAGE protein inhibitors for treatment of Alzheimers disease using ML.^3^ Their results of ML model selections correlated with the set of inhibitors selected using pharmacophore-based docking to the binding site. Maanaskumar and colleagues suggested the possible drugs for treatments of the COVID-19 cytokine storm based on ML models of inhibitors of top proteins involved in cytokine response. In their results the elucidated drug candidates were predicted to be more effective than the known compounds for all 5 of their targets.^4^ Gao and colleagues used ML with the following docking for selection of drugs for treating *Candida albicans* infections.^5^ The model has a cross-validation accuracy of 96.72%. Also, they note that five of the ML predicted drugs were found to inhibit *Candida* in experiments.^5^ ML has been extensively used to find new inhibitory candidates and has been shown to produce results.

## 2 Methods

### 2.1 Machine learning

**Fig. 1:**
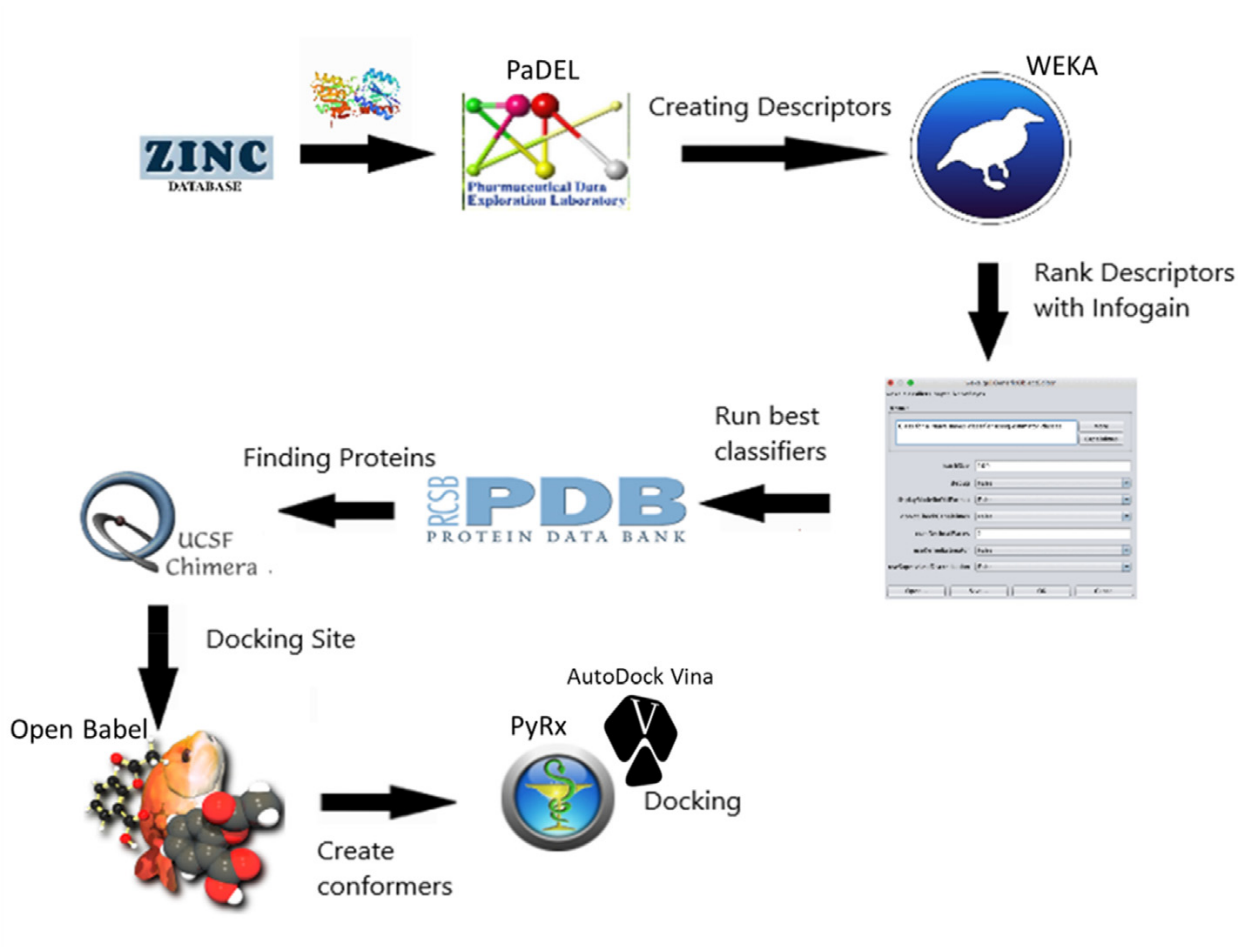
Methods. Compounds are data mined through the ZINC15 database, then the chemical descriptors were elucidated using PaDEL. These descriptors were ranked using InfoGain to find the most significant ones. Then the ML models were trained using the selected descriptors. These models were used for selection of the best fitting compounds from the FDA-approved drugs database. Then, using Open Babel program,^10^ we modeled 30 possible conformers of each of the selected compounds. Each of the conformers was docked to AR with the program PyRx.

The block-diagram of methods is presented in Fig. 1. To create the ML model of AR inhibitors we selected the known inhibitors from the Zinc15 database.^6^ Also, the training dataset included the random compounds selected from the FDA-approved drugs dataset in the ZINC15^6^ database. Database of the FDA-approved drugs contains around 1500 drugs that were approved by the US FDA for use in medical practice. We use this database to select a set of random drug compounds that are not linked to any specific disease. Drugs for this set are selected using a random-number generator without repetitions. This random set is used as a dataset of control in classification scheme including selected – disease-related compounds and random compounds. In total, 130 compounds were applied: 65 random compounds, and 65 known inhibitors. The criteria to select the known compounds were IC_50_ values less than 500 ​nM.

Preprocessing steps involved filtering chemical descriptors using InfoGain and normalization of data. First, we elucidated the compounds chemical descriptors using the PaDEL software,^7^ getting more than 1400 molecular descriptors. These descriptors were filtered using InfoGain function of WEKA (ML software).^8^ InfoGain is a short version of the name of the WEKA function InfoGainAttributeEval. It evaluates the worth of an attribute by measuring the information gain with respect to the class. Calculated through.

InfoGain(Class, Attribute) ​= ​H(Class) ​− ​H(Class | Attribute), the results give information about the difference between an attribute's value for a selected attribute and random attributes. If it is too small this attribute does not have a significant input in the ML model and can be removed. Descriptors that received a correlation value greater than 0.1 were kept: leaving a total of 459 descriptors remaining.

Then, we created the ML models of predicted AR inhibitors using different classifiers in WEKA. Models prepared with each classifier were tested using cross-validation. Each ML model is the unique combination of descriptors involvement, preprocessing steps, and classifiers selection with the parameters of each classifier adjusted to get maximum accuracy on the specific combination of input data and descriptors. So, one cannot just take any known ML models and use them for other purposes. Each ML models developed are unique.

All the classifiers available on WEKA were tested in our machine-learning model. Eventually Multilayer perceptron, LogitBoost, SMO, and MultiClassClassifierUpdateable were chosen because of their high accuracy rate. Furthermore, depending on the machine learning model, nominal data, or data that is descriptive rather than numeric, may be removed or added. Some models do not deal with different classes and are unable to account for them.

### 2.2 Docking

**Fig. 2:**
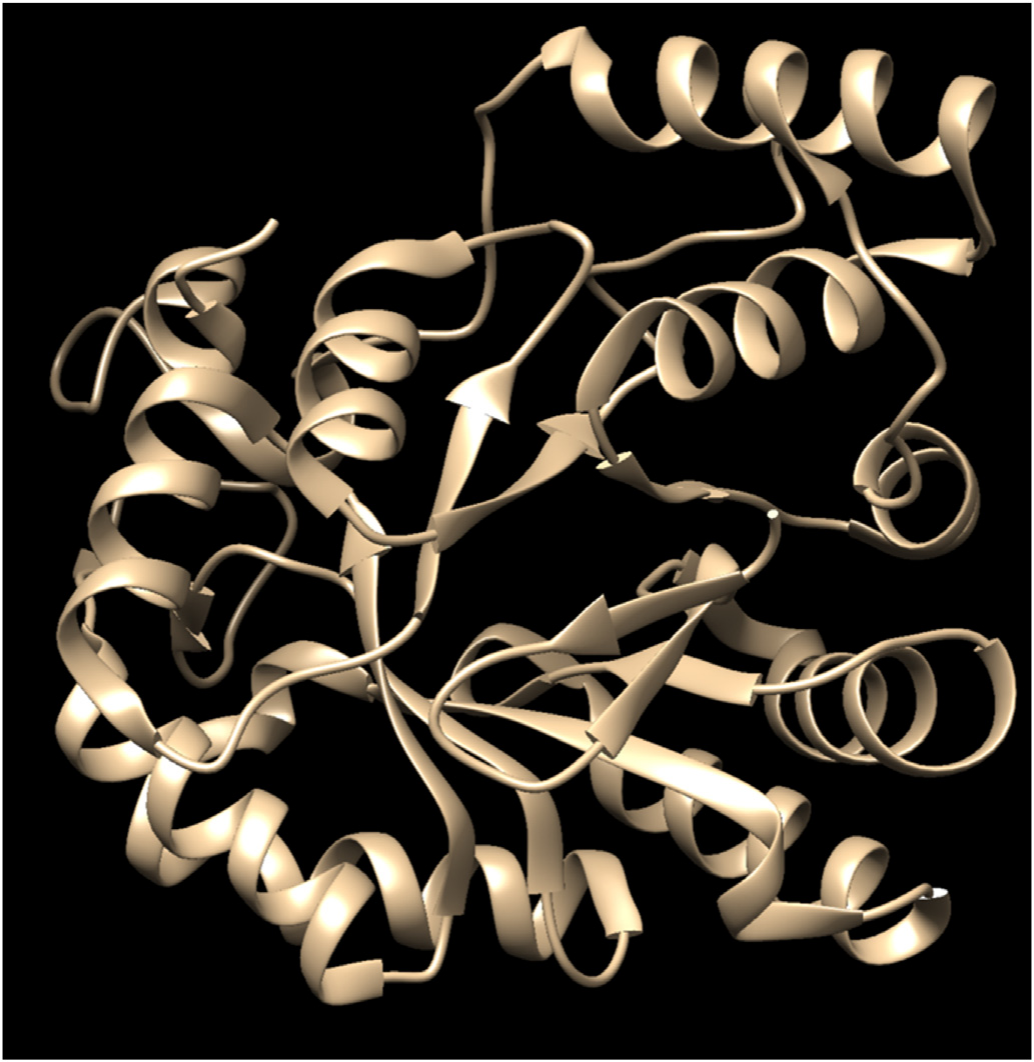
Human Aldose Reductase Structure (PDB ID: 2R24) complex with butanol removed. The protein had butanol removed from the complex to allow compounds to be docked in the main body.

Computational docking was used to test the selected ML compounds. Docking was performed with the program PyRx.^9^ The protein, PDB ID 2R24 (Fig. 2), which is Human Aldose Reductase structure, was used as the docking subject. The binding site was found through existing data on binding of known inhibitors. Through Open Babel,^10^ the PDB file was changed to a pdbqt file, which is the type of file required for docking, and was inputted into PyRx as a macromolecule (Fig. 2).

Selected with machine-learning, compounds then were docked to the AR. Thirty best-energy conformers were prepared for each compound using Open Babel subprogram. This program generates the large set of possible conformers for a compound taking in consideration the permitted angles of rotation and fixed lengths of the bonds.

Docking was then done using PyRx. The program AutoDock Vina,^11^ was used through PyRx, allowing us to set a binding site and dock each conformer separately.

## 3 Results

The cross-validation results for each classifier are shown below (Table 1), with the highest-scoring classifiers: Sequential minimal optimization (SMO), Multilayer perceptron, Logit Boost, J48, and MultiClassUpdateable. Out of these, Multilayer perceptron had an average true positive rate of 0.89, as well as MultiClassUpdateable, SMO, J48, and Logit Boost, and each had an accuracy score of 89.23%.

**Table 1:** True positive rate for best classifiers.

Classifier	TP Rate
Multilayer Perceptron	0.8923
SMO	0.8925
MultiClassClassifier	0.8920
LogitBoost	0.8920
J48	0.8925

The model was tested with a 90/10 split on cross validation. In the cross-validation the input dataset is split to two portions and one of them is not used for training but only for testing. Such calculations are done at least 10 times with every time different compound selected for split portions. There are not any similar known attempts to predict new AR inhibitors, so, no comparisons were made.

The prepared ML models were then applied for the dataset of 1355 FDA-approved drugs. As the result of testing, we selected 400 drug compounds having scores of 0.5 or higher for each of 4 selected top classifier-based models.

The performance of the ML models of AR inhibitors was then tested using the absolutely independent computer docking studies of the predicted compounds (30 possible conformations) to the AR molecule binding site.

**Fig. 3:**
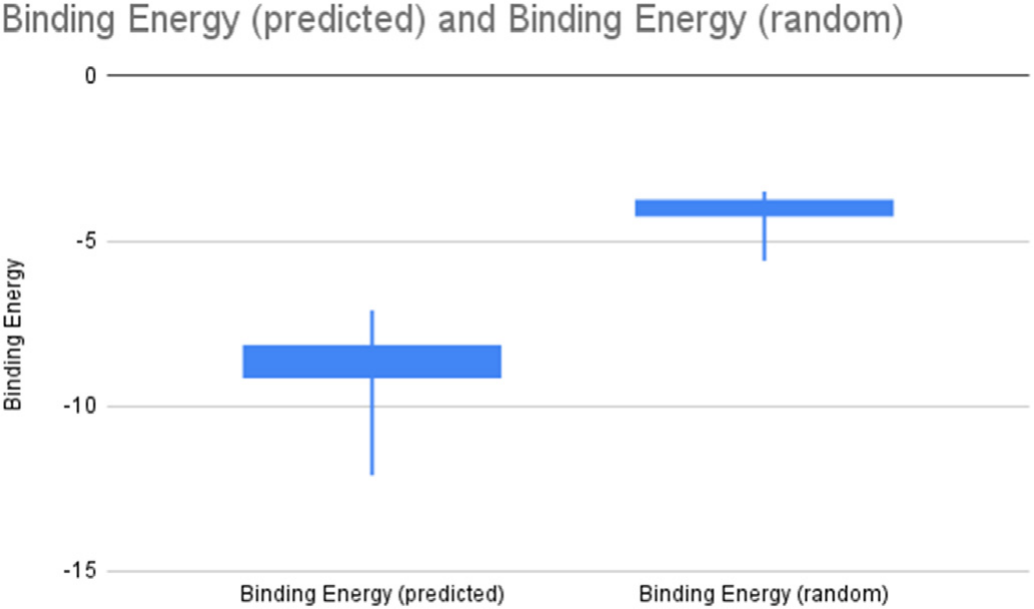
Box Plot of the binding energy of random compounds from ZINC database vs the binding energy of the predicted ML compounds docked using vina. The lower quartiles of the predicted compounds demonstrate that the ML compounds have stronger energies.

The binding energies of the ML-predicted versus random compounds show a stark contrast (Fig. 3). Compared to the random compounds binding energy, the energies of the predicted inhibitors (Table 2) are just more compact and lower, displaying that the ML model is quite accurate at finding suitable inhibitors for AR. The data for the random compounds have a max of −0.2, a 3rd quartile of −3.2, a median of −3.8, a 1st quartile of −4.2, and a min of −5.6. The data for the predicted compounds have a max of −2.5, a 3rd quartile of −7.1, a median of −8.2, a 3rd quartile of −9.2, and a min of −12.6. For the top 50 predicted compounds, binding energies are located below along with the correlation to the best Prediction Score (Table 2).

**Table 2:** Prediction-score–binding energy table. Higher predicted scores of compounds tend to have stronger binding energies.

Compounds	Prediction Score	Binding Energy (kcal/mol)
Pimozide	1	−12.6
ZINC000100013500	1	−12.5
Zafirlukast	1	−12.4
Bexarotene	1	−12.4
Altabax	1	−12.1
Nilotinib	1	−12.0
Fexofenadine	1	−11.9
Iloprost (ZINC000100052691)	1	−11.9
Ciclesonide	1	−11.8
Glipizide	1	−11.8
Droperidol	1	−11.8
Sqv (ZINC000026664090)	1	−11.8
Glibenclamide	1	−11.7
Ventavis (ZINC000100052685)	1	−11.7
Pioglitazone	1	−11.6
Belinostat	1	−11.6
Ziprasidone	1	−11.6
Iloprost (ZINC000100052681)	1	−11.6
Saquinavir	1	−11.6
ZINC000001999441	1	−11.5
Aripiprazole	1	−11.5
Lumacaftor	1	−11.4
Fulvestrant	1	−11.4
Olaparib	1	−11.4
Samsca	1	−11.4
ZINC000005844788	1	−11.4
Eltrombopag	1	−11.4
Vismodegib	1	−11.4
ZINC000100070954	1	−11.3
Axitinib	1	−11.3
Drospirenone	1	−11.3
Travopost	1	−11.3
Vemurafenib	1	−11.3
Dolutegravir	1	−11.2
ZINC000204073689	1	−11.1
Calcipotriol	1	−11.1
Sqv (ZINC000029416466)	1	−11.0
Ting	1	−10.9
ZINC000222731806	1	−10.9
Oxistal	1	−10.7
Iloprost (ZINC000100052688)	1	−10.7
Ezetimibe	1	−10.7
Riociguat	1	−10.6
Pioglitazone	1	−10.6
Cobimetinib	1	−10.6
Nebivolol	1	−10.6
Vorapaxar	1	−10.6
Glimepride	1	−10.6
Rosiglitazone	1	−10.6
Azulfidine	1	−10.6

**Fig. 4:**
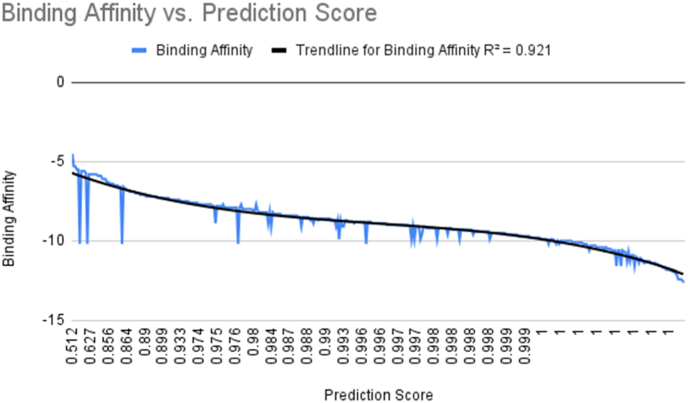
The trendline of the points using the binding energies of predicted ML energies vs. the prediction scores from the classifiers. There is a correlation coefficient of 0.959 between the binding energies and the prediction scores, demonstrating that the higher the prediction score, the stronger the binding energy. The r-squared value of the datapoints is 0.921, indicating very little variance within the data.

This table includes only the compounds having the prediction score 1. These compounds have energies better than −10.6 ​kcal/mol. The binding energies of the compounds having the prediction score less than 1 is presented in Fig. 4. The close correlation of Prediction Score and Binding energy shows the validity of this type of ML prediction method.

Comparing the computational docking binding energies versus the prediction score given by the ML models correlates very closely to one another, as shown in Fig. 4. The trendline gives an r^2^ value of 0.921, which is a befitting accuracy considering there are a few outliers that could be accounted for.

## 4 Discussion

When the possible AR inhibitors were selected, it was not assumed that they would be already known drugs that are used in the diabetes or diabetic cataract treatment. However, we found that 7 of the top 50 predicted inhibitors are known cataract drugs. Additionally, 5 of the top 50 predicted inhibitors are known AR inhibitors. Since the known AR inhibitors were found within a predicted set of possible AR inhibitors, it may demonstrate the strength of the machine-learning model and point that the remaining top score compounds may also be real AR inhibitors.

Several drugs, which were predicted with the ML model, have been tested as a possible treatment for cataracts, rosuvastatin, with a binding affinity of −10, has been extensively studied for treatment of cataract. The drug group of statins have been found to have a protective effect in "preventing cataracts, [especially] in younger patients and with longer duration of follow-up".^12^ The follow-up meta-analysis concurred with this result and "indicated a 19% decrease in cataract among statin users".^13^ Diosmetin with a binding affinity of −10.2 is another promising drug that was predicted with our ML model and docking. In a 2022 study, it was found that "by targeting MEK2 and reducing oxidative stress-induced MAPK pathway activation, diosmetin helps to protect lens epithelial cells against H_2_O_2_ and UVB-induced damage, suggesting diosmetin as a potential candidate for cataract treatment".^14^ Continued research discovered that Diosmetin restored oxidative damage and prevented cataracts.^14^ It is quite possible that its inhibiting of AR plays a role in this effect. Authors hypothesized that "the pleiotropic effects of statins including effects on inflammation and oxidation may mediate a decrease in the rate of cataract formation".^15^ AR function is to mediate the oxidative stress and redox imbalances, so the meta-analysis' conclusion lines up with the ML model's conclusion. Ezetimibe, binding affinity −10.7, in conjunction with rosuvastatin is associated with a 44% lower incidence of cataracts. Itis found that this combination of drugs also has effects on inflammation and oxidation, key developments in diabetic cataracts.^15^ Isoproterenol—another drug predicted in our ML and docking search. It was shown in experimental studies that it "virtually abolished cataract formation".^16^ Aripiprazole and eltrombopag, the most promising predicted by ML and docking candidates with a binding affinity of −11.5 and −11.4 respectively, were found to have no impact on development of non-diabetic cataracts, however their effects on diabetic cataracts are unknown.^17^ Olaparib, with a binding affinity of −11.4, was found to protect retinal cells from oxidative stress, which is involved in diabetic cataracts development.^18^ This falls in line with the predicted results. Rosiglitazone also seems to be a promising candidate with a binding affinity of −10.6. It is used in diabetic medicine, although its known up to date mechanism targets a different pathway from the polyol pathway.

Limitations within the experiment include lack of experimental data and aldose reductase inhibitors. In general, not a lot of existing drugs have been tested for AR. It is a well-studied protein, but even it has a lack of data available in public sources. For well-studied proteins, our method can be used to elucidate more novel inhibitors. There are also known problems in the ML models. Because specific classes of inhibitors of AR are studied more frequently, they tend to take up a higher margin of the ML model, creating some biases in results. It is a known problem of all ML methods of supervised learning.

These results demonstrate that using ML and docking can be an effective method to predict AR inhibitors. With future testing on these compounds for possible side effects and effectiveness on inhibiting AR, these predicted compounds may become an effective and safe way to treat diabetic cataracts. Although the ML models did not have an accuracy as high as expected and the InfoGain filtration procedure also had issues in extracting every significant characteristic involving binding to AR, overfitting or underfitting it, these issues could be fixed and did not have a major influence over the final results. Using the current strategy, more inhibitors may be found that could have less side effects or higher effectiveness. This approach may also be effective for finding inhibitors of other proteins that cause diseases.

## Study approval

This study does not need any approval.

## Author contributions

TC conducted computational docking and wrote the article, RC and AY selected the inhibitors and descriptors for the ML model and developed the ML model, VLK and IFT proposed the concept of the project and participated in the ML model development and computational docking studies, edited the article. All authors reviewed the results and approved the final version of the manuscript.

## Funding

This research did not receive any specific grant from funding agencies in the public, commercial, or not-for-profit sectors.

## Declaration of competing interest

The authors of the article Search of inhibitors of aldose reductase for treatment of diabetic cataracts using machine learning declare that they have no known competing financial interests or personal relationships that could have appeared to influence the work reported in this paper.
